# Leocarpinolide B Attenuates Collagen Type II-Induced Arthritis by Inhibiting DNA Binding Activity of NF-κB

**DOI:** 10.3390/molecules28104241

**Published:** 2023-05-22

**Authors:** Ke-Gang Linghu, Guan-Ding Zhao, Dai-Yan Zhang, Shi-Hang Xiong, Guo-Ping Wu, Li-Yu Shen, Wen-Qing Cui, Tian Zhang, Yuan-Jia Hu, Bing Guo, Xiang-Chun Shen, Hua Yu

**Affiliations:** 1 State Key Laboratory of Quality Research in Chinese Medicine, Institute of Chinese Medical Sciences, University of Macau, Macao SAR, China; yb77507@um.edu.mo (K.-G.L.); yc07513@um.edu.mo (G.-D.Z.); yc07528@um.edu.mo (D.-Y.Z.); yc17540@um.edu.mo (S.-H.X.); yc27538@um.edu.mo (G.-P.W.); mc05833@um.edu.mo (L.-Y.S.); wenqingcui@um.edu.mo (W.-Q.C.); yb67520@um.edu.mo (T.Z.); yuanjiahu@um.edu.mo (Y.-J.H.); 2 The Key Laboratory of Optimal Utilization of Natural Medicine Resources, School of Pharmaceutical Sciences, Guizhou Medical University, Guiyang 550025, China; 3 Guizhou Provincial Key Laboratory of Pathogenesis and Drug Research on Common Chronic Diseases, Guizhou Medical University, Guiyang 550025, China; huguobingbs@gmail.com

**Keywords:** leocarpinolide B, sesquiterpene lactone, rheumatoid arthritis, NF-κB p65, synovitis

## Abstract

Rheumatoid arthritis (RA) is a chronic autoimmune disease triggered by a cascading inflammatory response. Sigesbeckia Herba (SH) has long been utilized as a traditional remedy to alleviate symptoms associated with rheumatism. Our previous study found that leocarpinolide B (LB), a sesquiterpene lactone isolated from the whole plant of SH, possesses potent a anti-inflammatory effect on macrophages. This study was designed to evaluate the therapeutic effects of LB on RA, and further investigate the underlying mechanisms. In collagen type II-induced arthritic mice, LB was demonstrated to decrease the production of autoimmune antibodies in serum and inflammatory cytokines in the joint muscles and recover the decreased regulatory T lymphocytes in spleen. Moreover, LB significantly suppressed the inflammatory infiltration, formation of pannus and bone erosion in the paw joints. In vitro testing showed that LB inhibited the proliferation, migration, invasion, and secretion of inflammatory cytokines in IL-1β-induced human synovial SW982 cells. Network pharmacology and molecular docking suggested NF-κB p65 could be the potential target of LB on RA treatment, subsequent experimental investigation confirmed that LB directly interacted with NF-κB p65 and reduced the DNA binding activity of NF-κB in synovial cells. In conclusion, LB significantly attenuated the collagen type II-induced arthritis, which was at least involved in the inhibition of DNA binding activity of NF-κB through a direct binding to NF-κB p65. These findings suggest that LB could be a valuable lead compound for developing anti-RA drugs.

## 1. Introduction

Rheumatoid arthritis (RA) is a chronic autoimmune disease that affects approximately 1% of the population worldwide [1]. It is characterized by inflammation and destruction of synovial joints, leading to pain, stiffness, and loss of function [2]. Synovial fibroblasts, which are a significant cellular component of the synovial membrane, play a critical role in the development of rheumatoid arthritis (RA). Synovial fibroblasts in RA are characterized by a transformed phenotype, with increased proliferation, migration, and resistance to apoptosis [2]. These cells produce a range of pro-inflammatory cytokines and chemokines, such as interleukin-8 (IL-8), IL-6, and monocyte chemoattractant protein-1 (MCP-1), that recruit and activate immune cells to the synovial tissue [3]. The activated synovial fibroblasts in RA also produce matrix metalloproteinases (MMPs) and other proteases, leading to the degradation of the extracellular matrix and ultimately to cartilage and bone destruction [4]. Recent studies have shown that synovial fibroblasts also contribute to the pathogenesis of RA through their interactions with immune cells, including T cells, B cells, and macrophages, thereby amplifying the inflammatory response and leading to the perpetuation of the disease [5]. Hence, suppressing the inflammatory state of synovial cells and the infiltration of macrophages to synovial tissue is critical to attenuate synovial tissue inflammation and ultimately improve patient outcomes.

Traditional therapies for RA, such as disease-modifying antirheumatic drugs (DMARDs) and biologic agents, are effective, but they can have significant side effects [6]. As such, there is a need for the development of new, more targeted therapies for RA. Sigesbeckia Herba (SH) is commonly used as a traditional medicine for the treatment of various inflammation-related diseases (especially for rheumatism) in China [7] and some other Asian countries [8]. Our previous study found that leocarpinolide B (LB), a sesquiterpene lactone isolated from the whole plant of SH, possesses potent anti-inflammatory activity and great potential for RA treatment [9]. Building upon these discoveries, our current study aimed to investigate the therapeutic effects of LB in mice with collagen type II-induced arthritis. Additionally, we sought to elucidate the underlying mechanisms by which LB exerts its anti-arthritic properties. Our study presented compelling evidence that LB exhibited direct targeting of NF-κB p65, leading to the inhibition of NF-κB’s DNA binding activity. As a result, LB effectively suppresses inflammation in synovial cells, leading to the alleviation of arthritic symptoms. These findings provide strong support for LB as a potential candidate for treating RA.

## 2. Results

### 2.1. LB Reduced IL-1β–Induced Secretion of Inflammatory Cytokines in SW982 Human Synovial Cells

**Figure 1:**
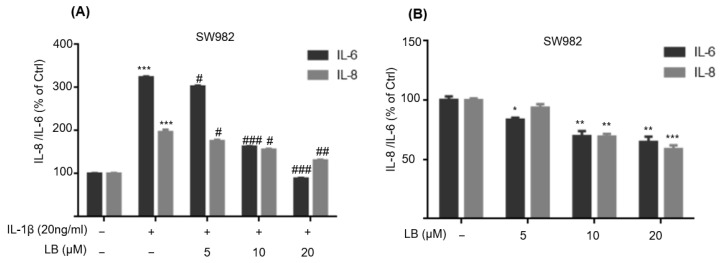
LB inhibited the IL-1β-induced secretion of inflammatory cytokines in SW982 synovial cells. (**A**) Cells were co-incubated with LB (5, 10, and 20 μM) and IL-1β (20 ng/mL) for 24 h, and the levels of IL-8 and IL-6 in the supernatant were measured. (**B**) Cells were treated with the indicated concentrations of LB for 24 h, then IL-8 and IL-6 in the culture medium were detected. Data were described as means ± SD (*n* = 3), * *p* < 0.05, ** *p* < 0.01, *** *p* < 0.001 versus control group; ^#^ *p* < 0.05, ^##^ *p* < 0.01, ^###^ *p* < 0.001 versus IL-1β alone group.

Previously, we determined that LB (≤20 μM) had no cytotoxicity, and LB showed potent anti-inflammatory effects on RAW264.7 macrophages [9]. To examine the effect of LB on synovial cell inflammation, SW982 cells were exposed to different concentrations of LB (5, 10, and 20 μM) for a duration of 24 h, both in the absence and presence of IL-1β (20 ng/mL). The levels of IL-6 and IL-8 were quantified using ELISA. As depicted in Figure 1A, the results demonstrated a dose-dependent inhibition of IL-6 and IL-8 by LB. Furthermore, when the cells were solely treated with LB for 24 h, the production of IL-6 and IL-8 was similarly dose-dependently attenuated (Figure 1B).

### 2.2. LB Demonstrated the Ability to Reduce IL-1β-Stimulated Proliferation, Migration, and Invasion of Human SW982 Synovial Cells

**Figure 2:**
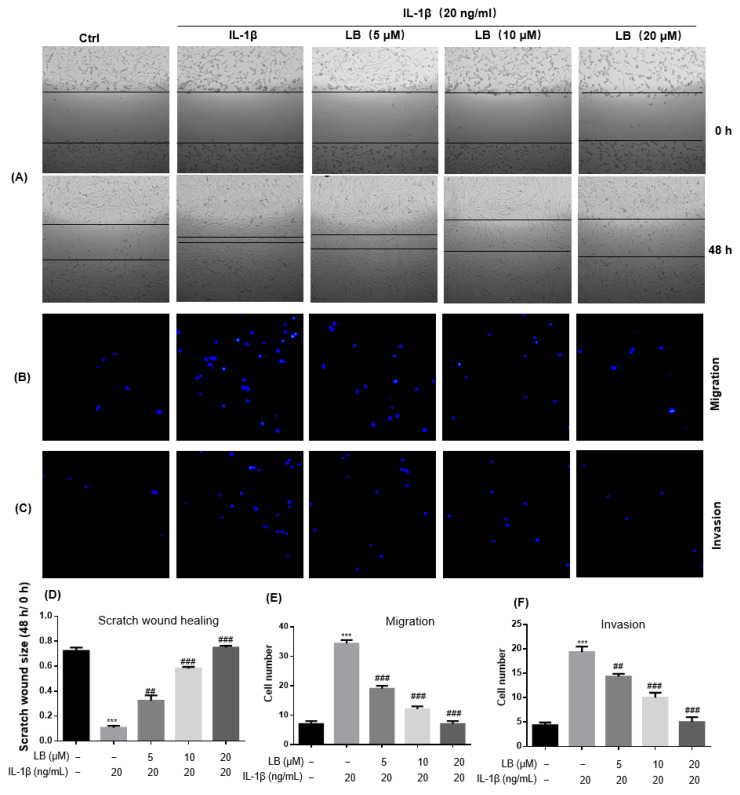
LB exhibited a notable attenuation of IL-1β-stimulated proliferation, migration, and invasion in human SW982 synovial cells. The experimental setup involved culturing cells in both 24-well plates (1 × 10^5^ cells/well) and 6-well plates (5 × 10^5^ cells/well) overnight. Subsequently, a scratch wound was created in each group using a 10-μL pipette tip in the 24-well plate. Cells were then treated with LB at concentrations of 5, 10, and 20 μM for 48 h in the presence of IL-1β at a concentration of 20 ng/mL. The healing of scratch wounds in the 24-well plate was observed using an inverted microscope, while the migration and invasion ability of cells in the 6-well plate were evaluated by using transwell plates. LB demonstrated significant inhibition of IL-1β-induced proliferation (**A**,**D**), migration (**B**,**E**), and invasion (**C**,**F**). The data were presented as means ± SD (*n* = 3), and statistical analysis indicated *** *p* < 0.001 versus the control group; ^##^ *p* < 0.01, ^###^ *p* < 0.001 versus the IL-1β alone group.

The scratch wound healing assay was evaluated in a 24-well plate. As shown in Figure 2A,D, the scratch wound size in IL-1β-induced cells was dramatically narrowed, while this proliferation could be reversed by LB in a dose-dependent manner. In order to assess the impact of LB on the migration and invasion of SW982 cells, a transwell chamber assay was employed. Cells were treated with LB in the presence of IL-1β, then transferred to the transwell chamber. The results revealed a dose-dependent inhibition of IL-1β-stimulated migration and invasion of SW982 synovial cells by LB, which are shown in Figure 2B,C,E,F.

### 2.3. LB Alleviated the Collagen Type II-Induced Arthritis in Mice

**Figure 3:**
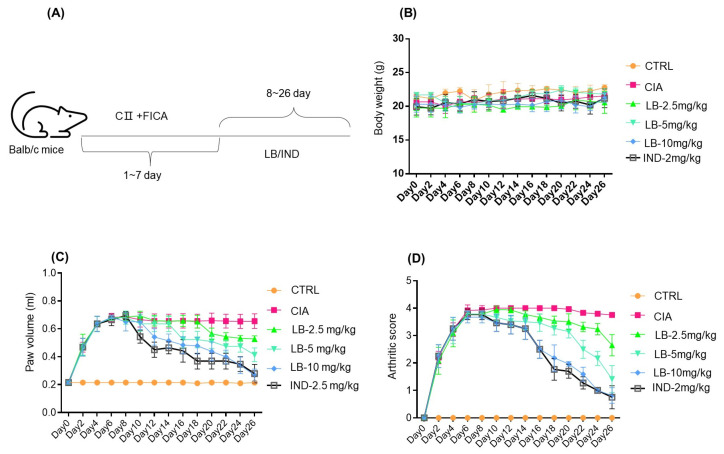
LB demonstrated its ability in alleviating collagen type II-induced arthritis (CIA). (**A**) The experimental procedure involved replicating the collagen-induced arthritis model in mice by administering emulsified bovine type II collagen in Freund’s incomplete adjuvant (FIA) once every 7 days. Based on body weight, mice were randomly divided into six groups (*n* = 6 each), including a Ctrl (vehicle) group, CIA (model) group, CIA-LB (2.5, 5.0, and 10.0 mg/kg) group, and CIA-IND (indomethacin, 2.5 mg/kg, positive control) group. Subsequently, the animals received intragastric administration of either saline or the specified drugs once daily for 19 consecutive days. (**B**) The body weight was detected during the experiment. (**C**) The hind paw volume (mL) was determined by using toe volume measuring instrument. (**D**) The arthritic score was evaluated by a mouse arthritis scoring system. Data are expressed as means ± SD (*n* = 6).

The collagen-induced mouse model of RA is widely accepted as a suitable model for assessing the efficacy of potential treatments for RA [10]. In light of the promising anti-inflammatory properties of LB on macrophages [9] and human synovial cells (Figure 1), we sought to investigate its therapeutic effect in the CIA mouse model (Figure 3A). Notably, no significant changes in body weight were observed during the experimental period, suggesting the absence of any LB-induced toxicity (Figure 3B). LB significantly alleviated the symptoms of CIA in mice. Specifically, LB administration progressively lowered foot swelling and arthritic scores as the duration of treatment increased (Figure 3C,D). Our findings provide evidence that LB has potential as a therapeutic agent for the treatment of RA.

### 2.4. LB Demonstrated a Moderating Effect on Inflammation-Related Endogenous Substances in Both Serum and Joint Muscle Tissue

**Figure 4:**
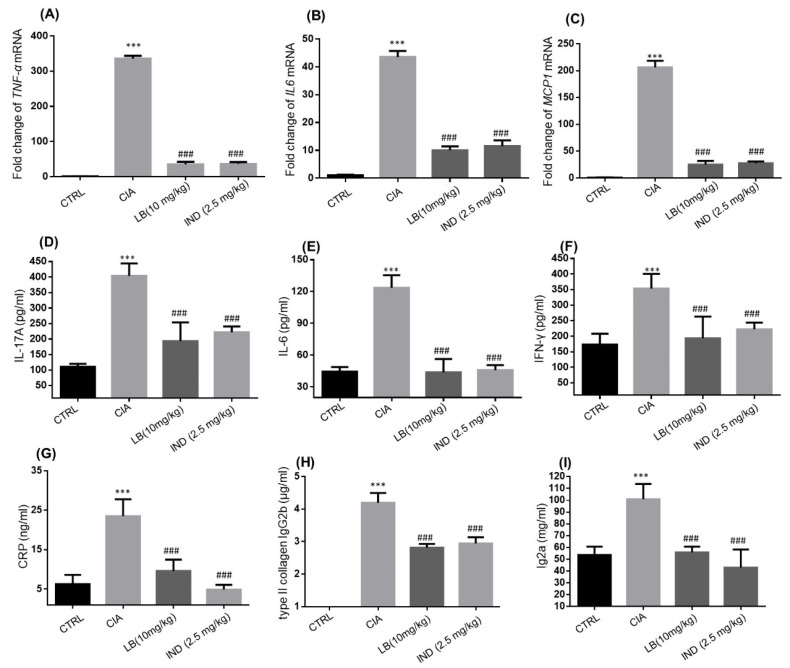
The impact of LB on inflammation-related endogenous substances in serum and joint muscle tissue was observed. Quantitative RT-PCR was performed to determine the mRNA levels of TNF-α (**A**), IL-6 (**B**), and MCP-1 (**C**) in joint muscle tissue. The levels of IL-17A (**D**), IL-6 (**E**), IFN-γ (**F**), CRP (**G**), type II collagen IgG2b (**H**) and Ig2a (**I**) in serum were determined by ELISA. Data are expressed as means ± SD (*n* = 6), *** *p* < 0.001 versus control group; ^###^ *p* < 0.001 versus CIA alone group.

As depicted in Figure 4A–C, LB (10 mg/kg) was demonstrated to significantly downregulate mRNA levels of inflammatory cytokines, including TNF-α, IL-6 and MCP-1 in the joint muscle of CIA mice. Furthermore, LB (10 mg/kg) significantly suppressed the expression levels of IL-17A (Figure 4D), IL-6 (Figure 4E), and IFN-γ (Figure 4F) in the serum of CIA mice, as demonstrated by ELISA results. Additionally, LB significantly reduced the levels of C-reactive protein (CRP) and autoantibodies type II collagen IgG2b and Ig2a in the serum of CIA mice (Figure 4G–I).

### 2.5. LB Reduced the Spleen Index and Increased CD4^+^FOXP3^+^ Cells in CIA Mice

**Figure 5:**
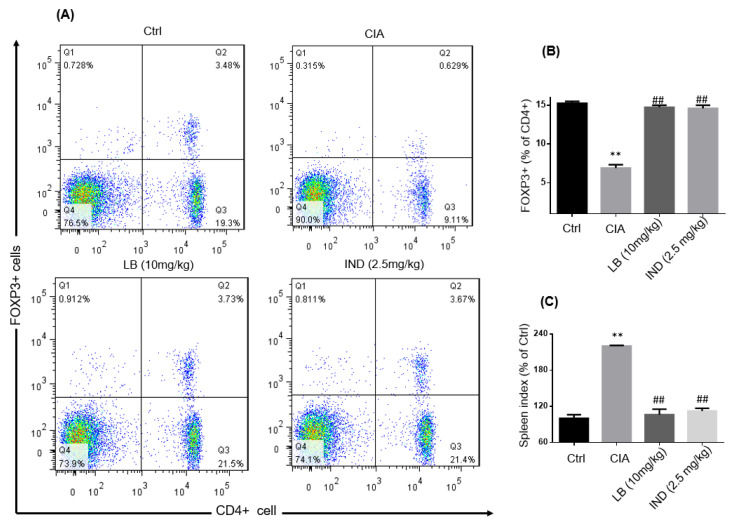
LB increased the level of CD4^+^FOXP3^+^ cells in spleen and decreased the spleen index in CIA mice. The CD4^+^FOXP3^+^ T cells proportion in spleen of CIA mice was evaluated by flow cytometry plots (**A**). The percentage of FOXP3^+^ in CD4^+^ T cells in spleen (**B**). The spleen index in different treatment groups (**C**). Data are expressed as means ± SD (*n* = 6), ** *p* < 0.01 versus control group; ^##^ *p* < 0.001 versus CIA alone group.

The unbalanced expression of T lymphocytes is an important cause of rheumatoid arthritis and has become a key indicator for evaluating RA. CD4^+^FOXP3^+^ T lymphocytes, also referred to as regulatory T cells (Tregs), play a critical role in immune regulation and are involved in suppressing immune responses and maintaining immune tolerance. Based on the previous discovery that the extract of SH has a regulatory effect on Tregs [11], the regulatory effect of LB on Tregs in spleen was further analyzed. LB was found to restore the proportion of Tregs in the spleen of CIA mice (Figure 5A,B). Additionally, LB reduced the increased spleen index in the CIA mice (Figure 5C). These results suggest that the therapeutic effect of LB on rheumatoid arthritis may involve the restoration of immune function.

### 2.6. LB Exhibited the Ability to Attenuate Both the Radiological and Pathological Features Observed in the Hind Paw Joints of Mice

**Figure 6:**
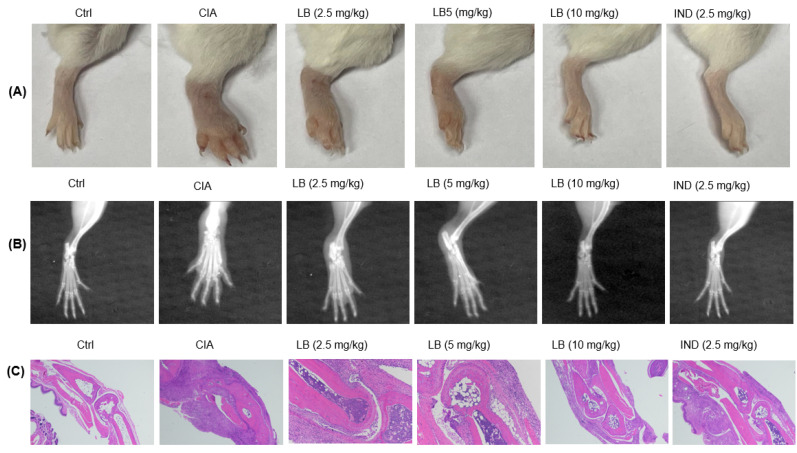
Photograph (**A**) and X-ray image (**B**) of the hind paw, histopathological evaluation of toe joints (**C**) by hematoxylin and eosin staining.

As depicted in Figure 6, in CIA mice, oral administration of LB effectively alleviated hind paw swelling induced by CII (A), and mitigated joint structural damage (B). Depicted in the histopathological images (C), in the Ctrl group of mice, a complete articular cavity at the toe joint was observed. However, in the CIA group, severe synovial hyperplasia characterized by the formation of pannus was observed, leading to the narrowing of the articular cavity, cartilage defects, and bone erosion. LB treatment effectively alleviated the abnormal proliferation of synovial cells and reduced the infiltration of inflammatory cells in the affected joints. As a result, LB exhibited a significant therapeutic effect in relieving the CII-induced damage to both bone and cartilage.

### 2.7. NF-κB p65 (RELA) Was Predicated to Be the Potential Target of LB on RA Treatment

**Figure 7:**
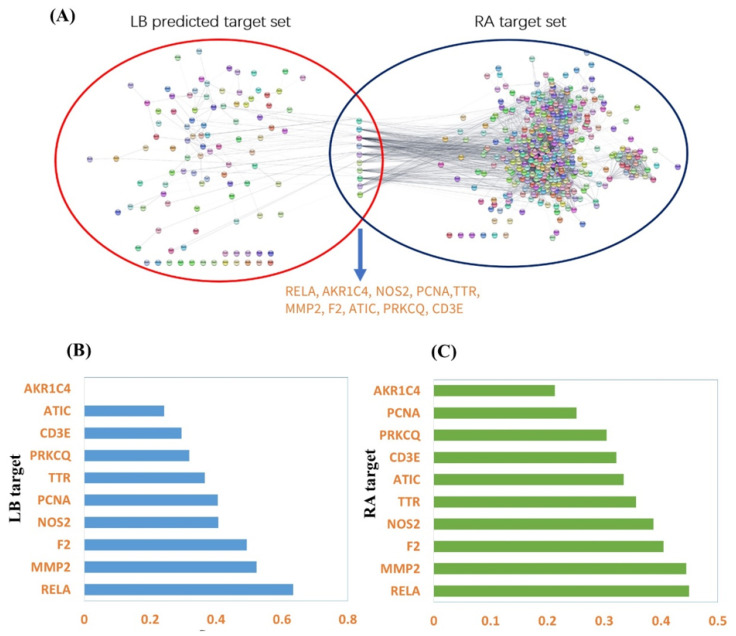
Shared targets of LB in RA targets. Ten shared targets of LB in RA targets (**A**). The score of each target in LB targets PPI network (**B**) and RA targets PPI network (**C**).

Above data confirmed that LB, as a potent active compound from SH herb, showed significant potential for RA treatment. We then predicted the potential targets of LB on RA treatment. Through the PharmMapper platform, after screening, we obtained a total of 64 drug targets and 65 ligand targets; through the SEA platform, we obtained a total of 1 target, RELA. After taking the union and removing the duplicates, the total targets were 114. Through the Drugbank database, a total of 175 drug-related targets were obtained; through the OMIM database, 21 phenotype-related targets were obtained; through the KEGG database, 93 targets in the pathway hsa05323 were obtained, and 181 targets were obtained from the CTD database. After taking the intersection and de-duplication, the total targets were 426. Finally, we found a total of 10 targets shared by the two target sets shown in Figure 7A. According to Figure 7B,C, RELA (NF-κB p65) shows a higher score in both LB targets and RA targets, which suggested NF-κB p65 could be a potential interaction node between the LB and RA.

### 2.8. LB Exhibited a Good Interaction with NF-κB p65 through Two Hydrogen Bonds

**Figure 8:**
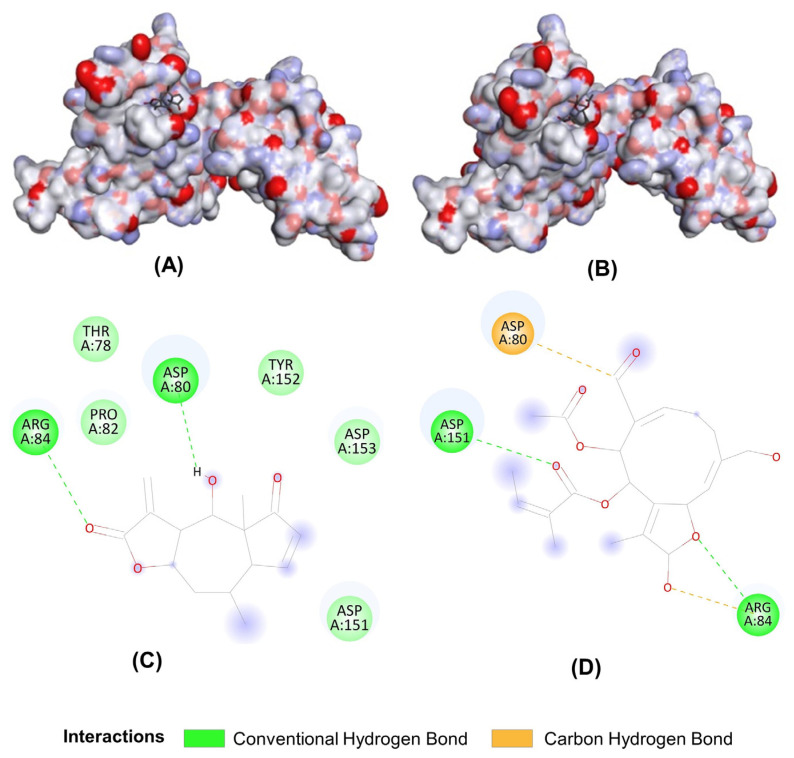
Molecular docking between LB and NF-κB p65. 3D binding modes of Helenanin (**A**) and LB (**B**) to protein NF-κB p65. 2D binding modes of Helenanin (**C**) and LB (**D**) to protein NF-κB p65.

Helenalin, with a similar structure to LB, has been reported to selectively inhibit transcription factor NF-κB by directly targeting NF-κB p65. Therefore, virtual molecular docking was conducted to compare the binding potency of LB and Helenalin with NF-κB p65. As shown in Figure 8, the results showed that both LB and Helenanin interacted with NF-κB p65 through the Conventional Hydrogen Bond or Carbon Hydrogen Bond; after calculating through the software, the binding score of LB and NF-κB p65 was −6.7 kcal/mol, and Helenanin was −5.8 kcal/mol, indicating that the binding effect of LB and NF-κB p65 is stronger.

### 2.9. LB Inhibited NF-κB DNA Binding Activity by Directly Targeting NF-κB p65

**Figure 9:**
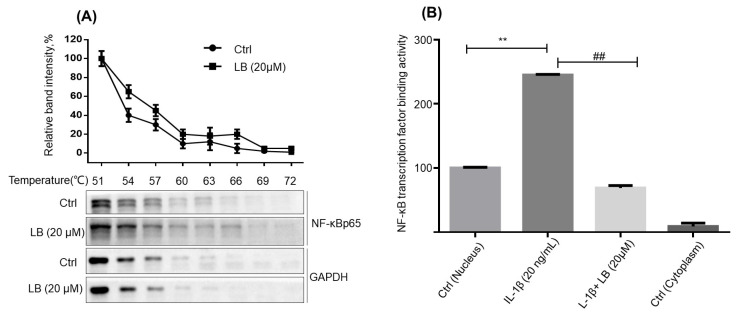
LB inhibited DNA binding activity of NF-κB through direct targeting of NF-κB p65. (**A**) Cellular thermal shift assay suggested the interaction between LB and NF-κB p65. SW982 synovial cells lysates were incubated with LB (20 μM) at 4 °C overnight, then 50 μL cell lysates (2 μg protein/μL) were transferred to new tubes and heated for 2.5 min for each tube at different temperatures (51–72 °C) using a thermal mixer, then the NF-κB p65 in the supernatants were detected by western blot (*n* = 3). (**B**) LB exhibited a significant inhibition on DNA binding activity of NF-κB. SW982 synovial cells were seeded on a 60 mm culture dish and allowed to adhere overnight. The cells were then treated with LB at a concentration of 20 μM for 1 h prior to stimulation with IL-1β at a concentration of 2 ng/mL for 2 h. Nuclear and cytoplasmic proteins in the cells were extracted following the procedures in the Transcription Factor Assay Kit, and NF-κB transcription factor binding activity were detected. Data are expressed as means ± SD (*n* = 5), ** *p* < 0.01 versus control group; ^##^ *p* < 0.001 versus CIA alone group.

To confirm the direct interaction between LB and NF-κB p65, a cellular thermal shift assay (CETSA) was performed. The results suggest that LB has the potential to directly target NF-κB p65, as it increased the protein thermostability of NF-κB p65. (Figure 9A). Guido Lyß reported that Helenanin selectively inhibits NF-κB DNA binding activity by directly targeting NF-κB p65. Given a stronger binding potency between LB and NF-κB p65 than between Helenanin and NF-κB p65, we further examined the effects of LB to NF-κB DNA binding activity. Interestingly, the results showed that LB significantly stopped the NF-κB DNA binding activity (Figure 9B). These results suggested that LB could inhibit NF-κB DNA binding activity through direct targeting of NF-κB p65.

## 3. Discussion

Rheumatoid arthritis (RA) is a chronic autoimmune inflammatory disease characterized by chronic inflammation, synovial hyperplasia, and cartilage and bone destruction, resulting in musculoskeletal deficits, decline in quality of life, reduced work capacity, and even shortened life expectancy [12,13].

Current treatments for RA are aimed at reducing inflammation and pain by using disease-modifying antirheumatic drugs (DMARDs) continuously, but they often have significant side effects and are not always effective in preventing disease progression [14]. The inquiry into prospective rheumatoid arthritis (RA) drug leads derived from natural sources has lately gained burgeoning scholarly and scientific attention [15,16,17,18].

We endeavor to examine the anti-arthritic impact and associated molecular mechanisms of Sigesbeckiae Herba (SH), a traditional medicinal herb utilized for the management of rheumatoid arthritis and joint infection [11,19,20]. Our findings revealed the discovery of a sesquiterpene compound, LB, which was extracted from SH and exhibited remarkable anti-inflammatory capacity on macrophages, indicating its potential for RA treatment [9]. This study was designed to evaluate the anti-arthritic effect of LB.

Macrophages and synovial cells are two kinds of important cells involved in the pathological process of RA, playing a major role in the initiation and perpetuation of destructive joint inflammation [5]. We had demonstrated the effects of LB on macrophages [9], thus, an evaluation on the synovial cells was performed in the current study. According to our experimental results in SW982 cells, LB treatment demonstrated a dose-dependent alleviation of IL-1β-induced inflammation, proliferation, migration, and invasion (Figure 1 and Figure 2). Therefore, we further investigated whether LB administration could attenuate the progression of RA in vivo. To simulate the pathological similarity to human RA, a collagen-induced arthritic mouse model was utilized. Administration of LB at a dose of 2.5–10 mg/kg significantly reduced RA symptoms, as evidenced by the reduction of paw swelling and arthritic score (Figure 3B,C). Moreover, LB exhibited a relatively safe profile on mice at tested doses, as body weight was not affected (Figure 3B). We further evaluated the effects of LB on systemic inflammation and joint protection and found that LB decreased the production of serum inflammatory cytokines, C-reactive protein (CRP), and autoantibodies (type II collagen IgG2b and Ig2a) (Figure 4G–I), restored the proportion of T lymphocytes in the spleen (Figure 5), and also attenuated joint damage (Figure 6C).

The abnormal signaling pathways have emerged as a crucial area of research in the diagnosing and treating of RA, including NF-κB, MAPK, WNT, PI3K/AKT, SYK, and JAK/STAT pathways [21,22,23,24]. NF-κB has been widely reported to be involved in the modulation of RA [25,26]. In almost all cell types, NF-kB composed of a p50 and p65 subunit is retained in an inactive cytoplasmic complex by binding to a third inhibitory subunit IκB [27]. A large variety of inflammatory conditions, such as bacterial and viral infection, as well as inflammatory cytokines, rapidly induce NF-κB activity. Active NF-κB p65 is released from the cytoplasmic complex and translocated to the nucleus, triggering the last but most critical step—binding with DNA to stimulate the transcription of inflammatory genes [28]. Our previous work suggested LB could regulate the Nrf2 and NF-κB signal pathways to ameliorate macrophage inflammation [9], but the direct target of LB on attenuating inflammation and RA has not been identified. Therefore, we predicated the targets of LB on RA treatment with network pharmacology combined with molecular docking. Results showed that NF-κB p65 (RELA) could be a critical target for anti-inflammation and anti-RA (Figure 7), and LB exhibited a better interaction than Helenalin (Figure 8), a sesquiterpene lactone anti-inflammatory inhibitor from Arnica by selectively inhibiting NF-κB p65 [29]. Importantly, the results of the CETSA (a powerful method for examining binding interactions in proteins [30]) confirmed that LB was able to bind to NF-κB p65 within cells, leading to an increase in the thermostability of the NF-κB p65 protein (Figure 9A). Moreover, LB inhibited the DNA binding activity of NF-κB p65 (Figure 9B), suggesting the possibility that LB could inhibit the DNA binding activity of NF-κB through direct targeting of NF-κB p65 to attenuate synovial inflammation in RA.

## 4. Materials and Methods

### 4.1. Chemicals and Reagents

Compound LB was isolated from dried Sigesbeckiae Herba, as previously reported [9]. Dimethyl sulfoxide (DMSO), 3-[4,5-Dimethyl-2-thiazolyl]-2,5-diphenyltetrazolium bromide (MTT) and lipopolysaccharides (LPS) from Escherichia coli O111:B4 were obtained from Sigma-Aldrich (St. Louis, MO, USA). Dulbecco’s modified eagle’s medium (DMEM), fetal bovine serum (FBS), phosphate-buffered saline (PBS), penicillin-streptomycin (10,000 U/mL, P/S), 0.25% Trypsin-EDTA (*w*/*v*), Nuclear and Cytoplasmic Protein Extraction Kit were obtained from Thermo Fisher Scientific (Waltham, MA, USA). ELISA kits were supplied by Neobioscience Technology Co., Ltd. (Shenzhen, China). Primary antibodies against NFκB-p65, GAPDH, and the secondary antibody were purchased from Cell Signaling Technology (Danvers, MA, USA).

### 4.2. Cell Culture

Human synovial cells (SW982) were purchased from the ATCC (American Type Culture Collection) and cultured in DMEM supplemented with 10% FBS and 1% P/S at a temperature of 37 °C in an environment with 95% humidity and 5% CO_2_. When the cells reached complete confluence, they were subcultured by using trypsinization, which involved treating the cells with 0.25% trypsin and 0.5 mM EDTA to detach them from the culture surface.

### 4.3. ELISA Assay for Culture Supernatant and Serum Sample

The concentrations of inflammatory cytokines in the culture supernatant, serum C-reactive protein (CRP) and autoimmune antibodies were quantified by using an ELISA kit following the manufacturer’s instructions and protocols.

### 4.4. Quantitative PCR

Total RNA in the joint muscle tissue were extracted using TRIzol Reagent, following the instructions provided by the manufacturer. Subsequently, the cDNA was synthesized from 1 μg RNA by using PrimeScript™ RT Reagent Kit (Takara (Kusatsu, Japan), # RR047A). Amplification reaction assay was performed with TB Green^®^ Premix Ex Taq™ (Takara, #RR420A) with gene specific primers. β-actin was used as the internal control.

### 4.5. Cellular Thermal Shift Assay

The cultured SW982 synovial cells were lysed with RIPA lysis buffer containing PMSF and protease inhibitor cocktail on ice for 10 min, then centrifuged (12,000× *g*, 10 min) at 4 °C. Cell lysates were incubated with or without 20 μM LB under shaking at 4 °C overnight. The protein concentration in the sample was quantified by using a BCA kit and subsequently adjusted to a concentration of 2 μg/μL in RIPA lysis buffer. 50 μL of cell lysates were aliquoted into new tubes and exposed to heat treatment for 2.5 min at different temperatures, ranging from 51 °C to 72 °C, by using a thermal mixer C (Eppendorf, Framingham, MA, USA). After centrifugation (12,000× *g* for 10 min), 40 μL of the supernatants were incubated with 10 μL 5× SDS-PAGE loading buffer at 95 °C for 10 min prior to western blotting assay.

### 4.6. Animals

Male Balb/c mice, aged 7–8 weeks, were housed in a standard animal laboratory environment. The mice were maintained under specific-pathogen-free conditions with access to a regular chow diet and water ad libitum. The housing conditions included a controlled temperature of 20–22 °C, a relative humidity of 50%, and a 12-h light/dark cycle. All experimental protocols (reference number: UMARE-029-2016) adhered to the guidelines set forth by the National Institutes of Health for the Care and Use of Laboratory Animals. These protocols were approved by the Animal Research Ethics Committee of the University of Macau, located in the Macau Special Administrative Region, China.

### 4.7. Drug Administration

The collagen-induced arthritis (CIA) model in mice was established by administering emulsified bovine type II collagen in Freund’s incomplete adjuvant, following the manufacturer’s recommended procedures (Chondrex, Inc., NE Redmond, WA, USA). Thereafter, mice were randomly divided into six groups (*n* = 6 each), including a Ctrl (vehicle) group, CIA (model) group, CIA-LB (2.5, 5.0, and 10.0 mg/kg) group, and CIA-IND (indomethacin, 2.5 mg/kg, positive control) group. Subsequently, the animals received intragastric administration of either saline or the specified drugs once daily for 19 consecutive days. The body weights of the animals were regularly monitored, and the clinical signs of arthritis were assessed and scored on a weekly basis following a previously described protocol [31]. Blood samples were collected from the orbits of the mice, and the serum was subsequently separated for further analysis by using ELISA. The mice were humanely sacrificed using CO_2_ inhalation. Tissues or organs were then carefully isolated on ice for specific experiments or stored at −80 °C for further analysis.

### 4.8. Radiographic and Histopathological Evaluation

On day-26, plain films of the hind paws were acquired using the IVIS Lumina XR imaging system Caliper, Hopkinton, MA, USA) to capture images for analysis. Then the hind paw was fixed and decalcified, as previously reported [11]. The decalcified joints were processed and embedded in paraffin, followed by sectioning into thin slices. These sections were then subjected to histopathological analysis using hematoxylin and eosin staining to examine the tissue structure and morphology [32].

### 4.9. Scratch Wound Healing Assay

SW982 cells were seeded onto a 24-well plate at a density of 1 × 10^5^ cells per well, allowing them to grow and form a confluent monolayer. A scratch wound was created in each group using a 10 μL pipette tip, and the unattached cells were subsequently removed by washing with PBS. The scratch wound was visually captured and recorded by a microscope. Following a 48-h co-treatment with LB (at concentrations of 5, 10, and 20.0 μM) in the presence of IL-1β (20 ng/mL), the healing of the scratch wound was quantitatively evaluated by measuring the recovered wound size using Image J Version 1.51.

### 4.10. Transwell Migration and Invasion Assays

To investigate the effects of LB on the migration and invasion of SW982 cells, a transwell chamber with an 8.0 μm pore size (Corning, Corning, NY, USA) was employed. After a 24-h co-treatment with LB (at concentrations of 5, 10, and 20.0 μM) in the presence of IL-1β (20 ng/mL), the cells were suspended in serum-free DMEM. Migration and invasion assays of synovial cells were performed as previously reported [11].

### 4.11. A Network Pharmacology Analysis Identified Potential Targets of LB for RA Treatment

#### 4.11.1. Prediction of Potential Targets of the LB

We drew the 3D structure diagram of the LB on ChemOffice Version 19.0 and converted it into mol2 format and SMILES format through the Open Babel Version 3.0.0. We imported the LB’s mol2 file into the PharmMapper analysis platform (http://www.lilab-ecust.cn/pharmmapper/, (accessed on 1 November 2020)) and obtained the top 300 “Ligandable” targets and “Druggable” targets. In addition, the Similarity Ensemble Analysis (SEA, http://sea.bkslab.org/, (accessed on 1 November 2020)) platform was used to predict potential targets of compound LB. Finally, we screened targets related to Homo sapiens through the Uniprot (https://www.uniprot.org/, (accessed on 1 November 2020)) database.

#### 4.11.2. Collection of Targets Related to Rheumatoid Arthritis (RA)

RA targets were selected from Drugbank (https://go.drugbank.com/, (accessed on 2 November 2020)), the Online Mendelian Inheritance in Man (OMIM, https://www.omim.org/, (accessed on 2 November 2020)), the Kyoto Encyclopedia of Genes and Genomes (KEGG, https://www.genome.jp/kegg/, (accessed on 5 November 2020)), and the Comparative Toxicogenomics Database (CTD, http://ctdbase.org/, (accessed on 5 November 2020)). In the Drugbank database, we collected the targets of drugs related to RA; in the OMIM library, we collected the targets related to the RA phenotype from the “PheneGene Graphics” part of the library; in the KEGG library, we found a description of the disease pathway “hsa05323: Rheumatoid arthritis” and collected related targets; in CTD, we found potential biomarkers/potential therapeutic targets related to RA.

#### 4.11.3. PPI Network Construction and Pathways Enrichment of LB and RA

Predicted targets of LB and the targets of RA were imported into the STRING (https://string-db.org/, (accessed on 5 November 2020)) database to visualize the PPI network, and they were, respectively, in the two networks, exhibiting the corresponding network parameters.

### 4.12. Molecular Docking of LB and RELA (NF-κB p65)

Three-dimensional crystal structure (PDBID: 2RAM) information of protein NF-κB p65 was downloaded from the PDB database and saved in PDB format. The positive small molecule ligands and macromolecular receptors (receptors) in the 3D crystal structure were separated through Discovery studio 4.5, then we used AutoDockTools to convert the positive ligands into PDBQT files, hydrogenate the receptors, add atoms, calculate the charge, save it as a PDBQT file, and find the best position and size of the receptor protein pocket through the Grid function of the software. We downloaded the molecule Helenanin (sdf file) in the PubChem database as a positive control and used the Open Babel Version 3.0.0 to convert it to a PDBQT file for docking. We used AutoDock Vina 1.1.2 as the docking software to calculate the binding of LB and the positive control to the protein NF-κB p65, respectively.

### 4.13. Flow Cytometry Analysis

The spleen was processed to obtain a single cell suspension by grinding and filtering through a 40 μm nylon cell strainer. The red blood cells in the single cell suspension were subsequently eliminated using a red blood cell lysate. The CD4^+^Foxp3^+^ cells population in the spleen were analyzed with flow cytometry, as previously reported [19].

### 4.14. NF-κB Transcription Factor Binding Activity Assay

SW982 synovial cells were seeded on 60 mm culture dish overnight, then the cells were treated with LB (20 μM) for 1 h prior to treatment with IL-1β (20 ng/mL) for 2 h. Nuclear and cytoplasmic proteins in the cells were extracted following the procedures in the Transcription Factor Assay Kit (Abcam, Cambridge, UK), and NF-κB transcription factor binding activity of NF-kB (p65) was detected accordingly.

### 4.15. Statistical Analysis

Statistical analyses were conducted using GraphPad Prism 6.0. Differences between groups were assessed using one-way ANOVA or two-way ANOVA, followed by Dunnett’s multiple comparisons test. The data were presented as means ± SD. Statistical significance was defined as *p* < 0.05.

## 5. Conclusions

To summarize, our study provides compelling evidence that LB has promising therapeutic potential for RA. We observed that LB effectively reduced IL-1β-induced arthritic inflammation in human synovial cells and mitigated RA progression in CIA mice. The anti-arthritic effects of LB were at least involved in the inhibition of the DNA binding activity of NF-κB through a direct binding to NF-κB p65. These findings disclose that LB is the important substance of efficacy in SH for treating RA, and suggest LB could be a valuable lead compound for developing anti-RA drugs.

## Data Availability

All datasets generated for this study are included in the article.

## Sample Availability

Samples of the compounds (Leocarpinolide B) are available from the authors.
